# The impact of sleep disorders of children with severe medical complexity on their caregivers’ sleep: a perspective from two pediatric palliative care centers

**DOI:** 10.1186/s13052-026-02231-8

**Published:** 2026-03-20

**Authors:** Filippo Pigani, Francesca Burlo, Francesca Peri, Marco Bolognani, Francesca Uez, Egidio Barbi, Lucia De Zen

**Affiliations:** 1 Department of Medicine, Surgery, and Health Sciences, University of Trieste, Trieste, Italy; 2 Pediatric Palliative Care and Pain Service, Institute for Maternal and Child Health - IRCCS Burlo Garofolo, Trieste, Italy; 3 Pediatric Palliative Care Network, Azienda Provinciale Per I Servizi Sanitari (APSS), Trento, Italy; 4 Pediatric Department, Institute for Maternal and Child Health - IRCCS Burlo Garofolo, Trieste, Italy

**Keywords:** Sleep, Pediatric palliative care, Caregivers, Cognitive impairment

## Abstract

**Purpose:**

This study aimed to investigate sleep patterns in children with severe medical complexity (CMC) eligible for pediatric palliative care (PPC) and to evaluate the specific impact of these patterns on their caregivers’ sleep quality and quantity.

**Methods:**

A multicenter descriptive and exploratory study was conducted across two PPC services in Italy, enrolling 15 children with high-complexity needs and 26 caregivers. Participants completed a dedicated questionnaire and a seven-day sleep diary to record data on sleep duration, nocturnal awakenings, medication use, and the caregivers’ estimated sleep loss. The relationship between the child’s sleep variables and the caregiver’s lost sleep hours was analyzed using Spearman rank-order correlation.

**Results:**

Children reported a median nocturnal sleep duration of eight hours, yet 86.6% experienced nocturnal awakenings. Conversely, caregivers reported a median sleep duration of six hours, estimating a median loss of two hours of rest per night. Statistical analysis revealed no significant correlation between the child’s total sleep duration and the caregiver’s sleep loss. However, a strong, statistically significant correlation was found between the number of the child’s nocturnal awakenings and the caregiver’s hours of sleep lost.

**Conclusion:**

Caregivers of CMC experience significant sleep deprivation, which is primarily driven by sleep fragmentation due to the child’s need for nocturnal assistance rather than the child’s total sleep duration. Even when children achieve a normal quantity of sleep, the frequent interruptions required for care severely compromise the parents’ ability to rest, highlighting the need for targeted support interventions.

## Abstract (highlights)

**What is known:**

Children with medical complexity (CMC) frequently experience disrupted sleep-wake cycles

Sleep disorders in these patients affect the quality of life of the entire family

**What is new:**

We examined direct correlations between the sleep quality of CMC and that of their caregivers

Caregivers’ sleep deprivation is mainly due to the child's sleep fragmentation rather than the child's total sleep duration

## Introduction

Children with medical complexity (CMC) often experience delayed sleep onset and disrupted sleep-wake cycles, increasing symptom burden and affecting their quality of life and that of their families [1–4].

Sleep disorders are particularly common among CMC, as those eligible for pediatric palliative care (PPC), with a prevalence of 50–80%, and are closely linked to a worse functional impairment [1–4].

Notably, many parents also suffer from sleep issues, irritability, stress, exhaustion, or headaches. However, data on the physical and psychological impact on CMC caregivers remain scarce.

Several tools assess sleep disorders, but only a few focus on CMC, as the Schlaffragebogen für Kinder mit Neurologischen und Anderen Komplexen Erkrankungen (SNAKE) questionnaire, which, however, is only limited to children with severe psychomotor impairment, or the more recent Sleep Screening for Children and Adolescents with Complex Chronic Conditions (SCAC), which includes larger populations [3, 5, 6].

Moreover, while studies often examine sleep disorders in patients, little is known about their impact on caregivers. Few recent studies assess caregivers’ sleep quality, but none directly compare it to the child’s one [6, 7].

This study aimed to investigate sleep disorders in children eligible for PPC and their impact on caregivers’ sleep.

## Methods

This multicenter descriptive study examined sleep quality in children and caregivers assisted by the PPC services in Trieste and Trento, Italy. Children and adolescents with chronic incurable diseases and high-complexity needs (based on the Assessment of Clinical and Care Needs in Pediatrics (ACCAPED) score, a validated tool to identify children with complex needs eligible to palliative care) were enrolled [8]. Those with malignancies or with poorly Italian speaking caregivers were excluded. This study was carried out from November to December 2024.

**Fig. 1:**
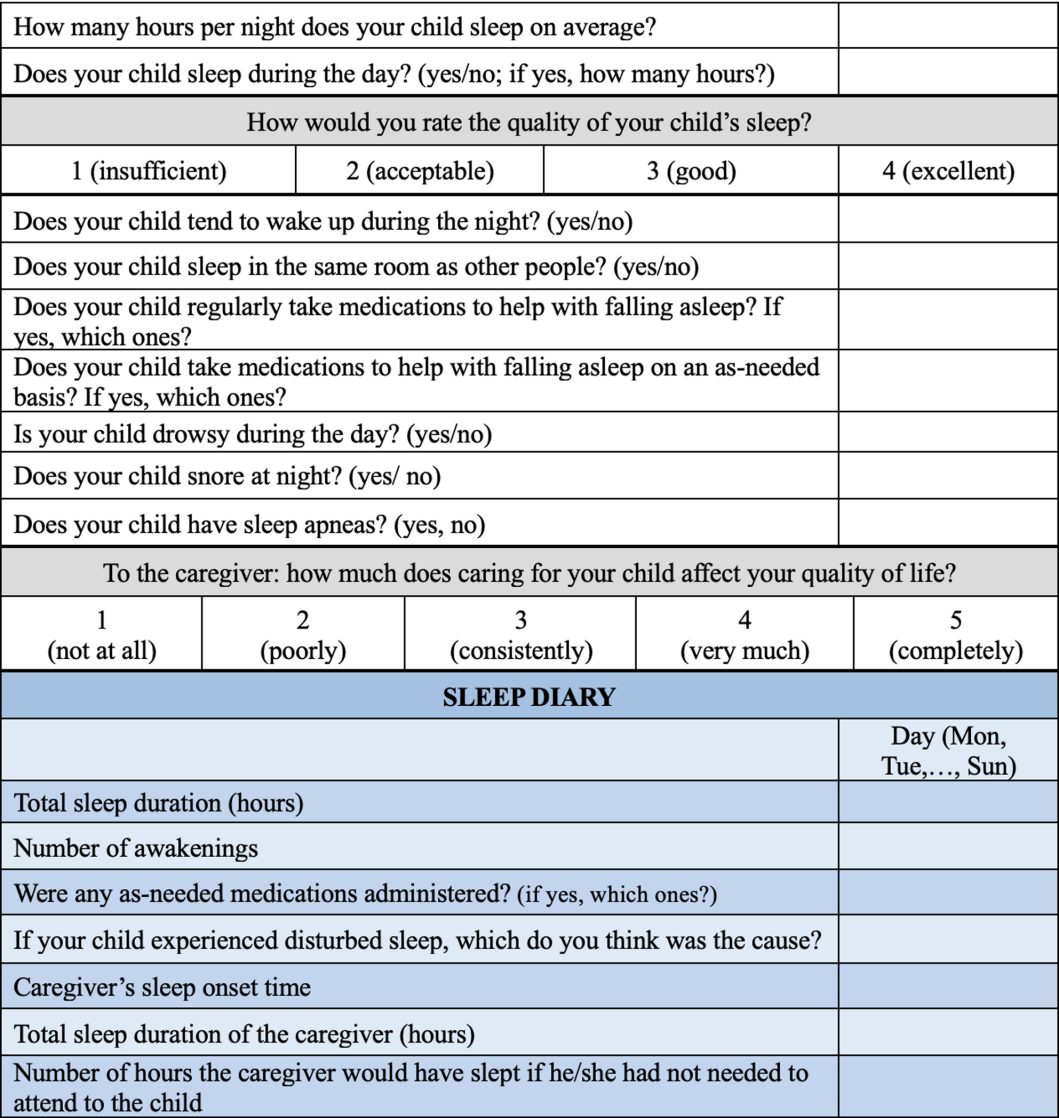
Questionnaire and a seven-day sleep diary assessing the child’s sleep duration, quality, medication use, daytime alertness, and the impact on the caregiver. In the seven-day sleep diary, caregivers recorded daily data on their child’s sleep and on their own sleep

Caregivers were asked to complete a questionnaire and a seven-day sleep diary (Fig. 1). The questionnaire was composed of two distinct sections. The first ten items, investigating the child’s sleep characteristics (including sleep duration, quality, nocturnal awakenings, medication use, and respiratory issues), were derived and adapted from the SNAKE questionnaire, as no validated tools are currently available in Italian. Conversely, the specific question addressing the direct impact of caregiving on the parent’s quality of life and the structure of the seven-day sleep diary were developed ad hoc for this study, as no validated tools covering these specific aspects were available. In the seven-day sleep diary, participants recorded daily data on sleep duration, the number and possible causes of awakenings, the need for “as-needed” medications administered, the caregiver’s sleep duration, and the estimated number of hours the caregiver would have slept if they had not to care for the child. The tools were preliminary tested on three caregivers, collecting their suggestions, before being finalized.

Data were collected in an anonymized database and analyzed using descriptive statistics. Categorical variables were described as absolute frequencies and percentages, while continuous variables were expressed as medians and interquartile ranges (IQR).

To assess the relationship between the child’s sleep variables (median nocturnal awakenings, median sleep duration) and the caregiver’s hours of sleep lost, the Spearman rank-order correlation (rho) was used. This non-parametric test was chosen due to the small sample size. The threshold for statistical significance was set at *p* < 0.05. Furthermore, the relationship between the clinical complexity score (ACCAPED) and sleep variables (child’s sleep duration, number of awakenings, and caregiver’s sleep loss) was investigated using the same statistical approach.

## Results

A total of 16 families were enrolled in the study. Among them, 15 completed and returned the questionnaire. Thus, 15 patients were enrolled in the study, with a median age of ten years (range 2–24, IQR 5.5).

Among the total sample, six patients (40%) were female. Eleven (73.3%) patients had a percutaneous endoscopic gastrostomy (PEG), and two (13.3%) had a tracheostomy. The mean ACCAPED scale of patients was 67.4. Considering the score of last question on the scale, which is strictly related to the child’s medical instability, four patients scored 0 (long-term survival is likely), seven scored 20 (upcoming events may threaten clinical stability), and four scored 50 (death within 12 months is likely).

Eleven (73.3%) children were classified at level 5 according to the GMFCS (Gross Motor Function Classification System).

A total of 26 caregivers (15 mothers and 11 fathers) completed the study questionnaires, with a median age of 51 years (range 20–59, IQR 8.5). In some cases, both parents participated, while in others, only one caregiver provided responses. No fathers participated without the corresponding mother from the couple also participating.

### Children’s sleep quality

The median duration of nighttime sleep was eight hours (range 6–10, IQR 2). Seven (46.6%) patients regularly took medication to sleep, while two (13.3%) used it only as needed. The most used sleep aid was melatonin (up to 10 mg), followed by nitrazepam, bromazepam, niaprazine, mirtazapine and cannabidiol.

Thirteen (86.6%) patients usually woke up during the night at least once. Eleven (73.3%) shared a room with other people, primarily caregivers. Six (40%) patients took daytime naps, with a median duration of one hour (range 1–3 h, IQR 45 min). Five (33.3%) patients experienced daytime sleepiness, even though four (80%) of them regularly napped.

Snoring was reported in two (13.3%) patients, and three (20%) experienced sleep apneas. The overall quality of sleep was rated as 3 on a scale from 1 (insufficient) to 4 (excellent). On a scale from 1 (no impact) to 5 (very high impact), the median impact of the child’s need for assistance on the caregiver’s sleep was 4.

### Seven-day sleep diary

From the seven-day sleep diaries, the median night sleep duration for patients was eight hours (range 0–12 h, IQR 2 h 15 min), with a median of two awakenings per night (range 0–4, IQR 1). Notably, the lower limit of the sleep duration range (0 h) corresponds to a single night of complete sleep deprivation reported for one child. Reported causes included suctioning needs, coughing, pain crises, and gastrointestinal issues like diarrhea or complications from nocturnal enteral feeding. In 21.8% of cases, caregivers could not identify the cause of awakening. Two patients (12%) required “as-needed” medications, mainly bromazepam and acetaminophen. Statistical analysis showed no significant correlation between the clinical complexity score (ACCAPED) and either the child’s sleep duration (rho = -0.16, *p* = 0.60) or the number of nocturnal awakenings (rho = -0.21, *p* = 0.49).

### Caregivers’ sleep quality

Regarding caregivers’ sleep, the median duration was six hours (range 0–9, IQR 1), while the median estimated number of hours they would have slept if they had not been required to care for the child was eight hours (range 6 h and 30 min – 9 h, IQR 1).

Statistical analysis revealed no significant correlation between the child’s total sleep duration and the caregiver’s hours of sleep lost (rho = -0.28, *p* = 0.404). Conversely, a strong, statistically significant positive correlation was found between the number of child’s nocturnal awakenings and the caregiver’s hours of sleep lost (rho = 0.72, *p* = 0.012). Furthermore, no statistically significant correlation was found between the ACCAPED score and the caregiver’s sleep loss (rho = -0.03, *p* = 0.92).

## Discussion

Given the exploratory nature of this study and the limited sample size, our findings should be regarded as preliminary and hypothesis-generating. The main purpose of this study was to evaluate the correlation between the sleep characteristics of children in palliative care and the sleep experienced by their caregivers. The main finding of this study is that caregivers’ sleep appears to be significantly compromised despite a reported reasonable amount of children’s sleep. Remarkably, while the children included in the sample reported an overall sleep duration within the normal range, their nights were characterized by frequent awakenings, requiring continuous parental assistance. This resulted in a marked reduction of caregivers’ sleep time, approximately two hours less than their estimated need. This discrepancy indicates that caregiving duties take precedence, suggesting that parents prioritize their child’s well-being while their own needs come second.

Moreover, statistical analysis demonstrated that the caregiver’s sleep loss was not related to the child’s total sleep duration but was significantly correlated with the number of nocturnal awakenings. This finding supports the hypothesis that caregiver sleep deprivation is primarily driven by the fragmentation of the child’s sleep and the need for interventions, rather than by the child’s total sleep quantity.

It must be acknowledged that the subjective estimation of sleep loss may be significantly influenced by caregiver ‘hypervigilance’ and anxiety regarding the child’s clinical stability. This psychological burden can lead parents to perceive severe sleep deprivation independently of the actual number of nocturnal awakenings.

The severity of the clinical condition, as measured by the ACCAPED score, did not correlate with the degree of sleep disruption. However, this finding must be interpreted with caution, as the small sample size of the cohort likely limited the statistical power to detect significant associations.

The occurrence of multiple and often unpredictable awakenings, not always attributable to identifiable causes, represents a critical factor leading to both sleep fragmentation and a perceived poor quality of rest. These findings are consistent with the existing literature, which highlights how caregivers of CMC are exposed to chronic sleep deprivation, with documented consequences on both physical and psychological well-being [9].

The correlation between the number of sleep hours reported by parents in the preliminary questionnaire, based on memory recall, and the sleep duration recorded through the sleep diary in this population demonstrates the parents’ ability to quantitatively represent their children’s sleep [10].

It is noteworthy that in several cases parents reported a good quality of sleep for their children, despite the frequent nocturnal interruptions. This discrepancy suggests that the use of sleep-promoting medications may improve the perception of children’s sleep, while not reducing the need for parental interventions during the night. Sleep-promoting medications are very common among CMC and are frequently adjusted according to the child’s response. While they may facilitate sleep onset or maintenance, they may not reduce the need for caregiver assistance, for example in relation to aspiration management or alarm checks [11]. However, this study did not compare nocturnal caregiver assistance between children receiving and not receiving sleep-promoting medications; therefore, this observation remains speculative.

A previous study on Rett-related syndrome patients demonstrated a discrepancy between the actual sleep quality of children with cognitive impairment and the parental reports regarding their children’s sleep quality [12]. Moreover, since it is evident that sleep quantity and quality are not necessarily proportional, this disparity becomes particularly important in a population in which sleep quality cannot be self-reported but can only be assessed through observational criteria assessed by an external observer, who may potentially be exposed to data loss, leading to the possibility of an underestimation [13].

This study has some limitations, starting from the small size sample and the clinical heterogeneity of the enrolled population. A further limitation lies in the absence of any objective sleep measurements (such as polysomnography). The subjective estimation of ‘lost sleep’ introduces a potential recall bias, as stress and anxiety may lead to overestimation. All data were collected through a parent-reported questionnaire, with the well-known potential risk of bias related to subjective perception. Moreover, this questionnaire was not validated into Italian. However, it was integrated with additional questions on child and caregiver’s sleep quality and a weekly sleep diary for real-life insights.

A further limit is that a qualitative approach was not considered while parents’ opinion could have been useful for a more thorough evaluation.

However, by integrating data from both parents and children, this approach could inform future studies to further explore caregiver sleep and related health conditions (e.g., hypertension, anxiety, depression).

In conclusion, our findings confirm that caregivers’ sleep is profoundly affected by the nocturnal care needs of their children, resulting in both reduced and fragmented rest. Future studies should incorporate objective sleep measures to more accurately describe the impact of caregiving on sleep and its long-term consequences on caregivers’ health. Further investigation is required into the sleep patterns of children in palliative care, where the scarcity of data and effective therapies remains a major critical issue for families.

## Conclusions

Within the limits of this exploratory analysis, these data suggest that despite children’s reasonable quality of sleep, parents’ sleep quality remains compromised. Factors beyond nocturnal awakenings, such as anxiety, fear of missing pain episodes, apneas, or seizures, may play a significant role. Sleep difficulties are often underestimated in parents’ population, highlighting the importance of incorporating objective assessment tools and qualitative tools as well. Future studies should investigate more thoroughly parents’ perspective.

## Data Availability

The data that support the findings of this study are available on request from the corresponding author.

## Abbreviations

ACCAPED: Assessment of Clinical and Care Needs in Pediatrics
CMC: Children with Medical Complexity
PPC: Pediatric Palliative Care
SCAC: Sleep Screening for Children and Adolescents with Complex Chronic Conditions
SNAKE: Schlaffragebogen für Kinder mit Neurologischen und Anderen Komplexen Erkrankungen
